# The role of pharmacological interventions in managing urological complications during pregnancy and childbirth: A review

**DOI:** 10.1097/MD.0000000000041381

**Published:** 2025-02-14

**Authors:** Isaac Edyedu, Okechukwu Paul-Chima Ugwu, Chinyere N. Ugwu, Esther Ugo Alum, Val Hyginus Udoka Eze, Mariam Basajja, Jovita Nnenna Ugwu, Fabian Chukwudi Ogenyi, Regina Idu Ejemot-Nwadiaro, Michael Ben Okon, Simeon Ikechukwu Egba, Daniel Ejim Uti, Patrick Maduabuchi Aja

**Affiliations:** a Faculty of Clinical Medicine Kampala International University, Kampala, Uganda; b Department of Publication and Extension, Kampala International University, Kampala, Uganda; c Health Care and Data Management Leiden University, Kampala, Uganda; d Department of Public Health, School of Allied Health Sciences, Kampala International University, Kampala, Uganda; e Directorate of Research, Innovation, Consultancy and Extension (RICE), Kampala International University, Kampala, Uganda; f Department of Biochemistry, Kampala International University, Kampala, Uganda.

**Keywords:** antibiotic therapy, interdisciplinary approach, pharmacological management, pregnancy, urinary tract infections, urological complications

## Abstract

Pregnancy leads to a number of structural and functional changes in the urinary system, which makes females susceptible to urological complications. This review aims to discuss the epidemiology, complications and prevention and management of urinary tract infections (UTIs), kidney stones and bladder dysfunction in pregnancy. UTIs are the most common urological problem presenting in 10% of pregnant women; Escherichia coli is the most common causative organism. If left untreated, UTIs lead to acute pyelonephritis which occurs in about 2% of pregnant women and which has serious consequences for both the mother and the baby. Kidney stones, although rare, are hazardous, occurring in 1 in 200 to 1 in 1500 pregnancies, and may cause obstructive uropathy, and aggravation of “labor-like” pain. Urological complications are frequent in pregnancy; bladder dysfunction alone has been documented to affect 50% of the pregnant women. Urological complications can have severe consequences when not properly managed including preterm labor and renal dysfunction. In order to have the best pharmacological care, safe use of antibiotics for UTIs is needed along with other measures for kidney stones. This review highlights the importance of a team approach to patient management to optimize outcome and touches briefly on some of the ethical dilemmas that may be encountered when drug therapy in pregnancy is being considered. Therefore, it is feasible to enhance the health of women and the fetus during this period through patient focused care and innovative interventions.

## 1. Introduction

Pregnancy is a unique phase of a woman’s life, characterized by multiple morphological and functional changes and this is very much true in the urinary tract.^[1]^ Such changes as ureteral dilation, increased renal plasma flow, and contribute to variation in bladder tone put pregnant women at risk of various urological complications.^[2]^ These include urinary tract infections, kidney stones, and bladder problems where each of them presents specific complications to the obstetric and fetal welfare.^[3]^ Minimal Access Surgery in Pregnancy Urological complications occur in up to 10% of pregnant women^[4]^ with urinary tract infection being the most common scoped. The hormonal changes inherent in pregnancy, especially increased levels of progesterone have a direct effect that results in the relaxation of the smooth muscles of the urinary tract and therefore urinary retention and incomplete voiding.^[5]^ The most common bacteria isolated in patients with these infections is Escherichia coli.^[6]^ If left untreated, the condition may progress to acute pyelonephritis, a severe complication affecting approximately 1% to 2% of pregnant women and has been linked with adverse birth outcomes including preterm delivery, low birth weight, and fetal mortality.^[7]^ These complications show the importance of proper screening and treatment of urinary tract infections (UTIs) as vital in prenatal care, which suggests that proper management of a patient’s condition is beneficial to patient outcomes.^[8]^ Kidney stones though less common than UTIs pose significant risks to the overall health during pregnancy with occurrence ranging from 1/200 to 1/1500.^[9]^ Changes in the calcium metabolism which are typical of pregnancy lead to the formation of the stones.^[10]^ These may cause severe pain, which may mimic labor and lead to obstructive uropathy, hence increasing the chances of infection as well as preterm labor.^[10]^ Preoperative management is usually conservative to safeguard maternal and fetal well-being because invasive procedures are discouraged unless necessary.^[10]^ Another issue is bladder dysfunctions, including urinary incontinence and overactive bladder (OAB), which affect up to half of the pregnant women.^[11]^ Another reason is the increased pressure from the enlarging uterus on the bladder making women feel the urgency and need to pass urine often.^[12]^ That is why these conditions, even though usually non-threatening, may entail a significant reduction in the quality of life during pregnancy. Basically, due to the negative impact of bladder dysfunction, there is a need to ensure that patients and clients receive adequate attention and management to ameliorate the situation.^[13,14]^ Management of these urological complications thus requires a mix of pharmacological and non-pharmacological measures.^[15]^ For example, the management of UTI continues to be directed by antibiotics, and first-line drugs like beta-lactams like amoxicillin and cephalexin are favored because of their safety profiles.^[16]^ The emergence of bacteria that are resistant to antibiotics calls for the use of culture and sensitivity tests in choosing the right treatment.^[17]^ Likewise, acute pyelonephritis should be managed with utmost intensity and often calls for hospitalization and intravenous use of antibiotics while kidney stones are best managed with conservative measures, and noninvasive methods being prioritized to ensure fetal safety.^[18]^ Considering the issues surrounding drug safety during pregnancy, it is important to follow the protocols provided by American College of Obstetricians and Gynecologists (ACOG) and the Centers for Disease Control and Prevention (CDC).^[19]^ These guidelines recommend the use of evidence-based safe medicines, especially during the first trimester as stated earlier. Moreover, new developments in pregnant women’s care stem from trends in the concept of individualization of the management, where decisions are made based on the patient’s characteristics and their condition, age in particular, and gestational age.^[19]^ The goal of this review is a narrative synthesis of the current state of knowledge on urological complications during pregnancy in terms of their incidence patterns, approaches to treatment, and effects on maternal and fetal outcomes. In line with this, the current review provides healthcare providers with information from the latest evidence and clinical practice guidelines on screening, diagnosing, and managing cases.^[20]^ Therefore, pointing to the fact that such patient management has multiple elements, the review also highlights the importance of collaboration in achieving the best results for pregnant women with urological diseases. It will also give an understanding of the literature present which will be useful in the clinical practice to enhance the care of pregnant women.

### 1.1. Rationale

Pregnancy causes significant alterations in anatomical and physiological structures of the lower urinary tract which lead to urological complications that may pose threats to the health of the mother and the unborn child.^[21]^ This paper recognizes the importance of understanding the incidence and the best ways of handling these conditions. As this paper is a narrative literature review, this study strives to mitigate gaps within the existing literature on best practices for the management of pregnant women through the identification of the most up-to-date evidence with which to arm healthcare providers. Furthermore, examining ethical issues and cross-disciplinary methods will guarantee the improved handling of the issues in question and result in better health for moms and their babies.

## 2. Methodology

### 2.1. Research design

This research used systematic review to find prevalence rates, management, and effects on mother and fetus of urological complications during pregnancy.

### 2.2. Literature search strategy

A comprehensive search was conducted using key databases: To this end, the databases used in this study include PubMed, Scopus, and Google Scholar. The following search terms and keywords were utilized: Complications related to the urinary system in pregnancy “urinary tract infections” “kidney stones” “bladder dysfunction” “pregnancy management” The data was filtered according to specific inclusion and exclusion criteria set before the study.

### 2.3. Data collection methods

Data were extracted from selected studies, focusing on: The incidence of urological complications. Pharmacological and non-pharmacological interventions for treatment of the above mentioned disorders. Health outcomes on the mother and the fetus. The extracted data was then arranged in a format suitable for analysis as presented below.

### 2.4. Inclusion criteria

Population: Research which was conducted on pregnant women with urological problems; UTIs are infection of the urinary system, which may affect any part of it. Kidney stones and Bladder dysfunction

Types of Studies: Research articles, guidelines, case reports and systematic reviews in English language from 2010 to the present.

Outcomes: Studies providing data on: Incidence rates, Management strategies, Maternal and fetal outcomes. Quality of life in connection with urological complications

### 2.5. Exclusion criteria

Non-Pregnant Populations: Previous researches produced on urological complications were conducted on non-pregnant patients or patients with other diseases.

Inaccessible Literature: Articles published in languages other than English or articles which are not accessible in full text.

Older Studies: The data collected in this research was from previous studies done before 2010 to make it relevant with today’s practice.

Irrelevant Topics: Those works that do not contain information concerning urological complications or are devoted to other diseases.

### 2.6. Strengths and limitations

Strengths: The use of recent literature makes the work relevant.

This paper aims to give a general view about the topic focusing on different urological complications.

Limitations: Possible publication bias since only peer reviewed articles were used in the analysis.

Due to the non-uniformity of study designs, and methodologies, the results may not be generalizable to other populations.

### 2.7. Ethical considerations

Because this study is a literature review, it does not fall under the category which requires ethical clearance. Nevertheless, utmost precaution was made to cite all the sources that were used in this research paper in order to avoid cases of plagiarism.

### 2.8. Analysis techniques

Findings from the selected studies were synthesized narratively, focusing on: Common themes and patterns. Evidence-based practices and the existing research gaps.

### 2.9. Research question

Which are the prevalence rates of urological complications during pregnancy and breastfeeding and what are the treatment options and health outcomes of affected.

### 2.10. Overview of urological complications during pregnancy

Any pregnant woman is at increased risk of developing various urological problems because pregnancy results in changes in the location and structure of the urinary system.^[21,22]^ These changes include ureteral dilation which increases the risk of the formation of kidney stones, increased renal plasma flow which increases the vulnerability of urinary tract infections and bladder that has a decreased tone which can lead to complications such as bladder dysfunction in Table 1.^[4,23]^ Knowledge of these problems and how to deal with them helps achieve good outcomes for the mother and baby. Urinary Tract Infections are the prevalent urological complication of pregnancy observed in 10% of pregnant females.^[23]^ Hormonal changes particularly increased progesterone cause relaxation of smooth muscles seen in the urinary tract hence leading to urinary retention and incomplete emptying of the bladder.^[24]^ This leads to the proliferation of bacteria particularly Escherichia coli which is the most prevalent in cases of pregnant mothers with UTI. If not managed, the UTIs can complicate into acute pyelonephritis, a severe kidney infection that occurs in 2% of pregnant women and increases chances of preterm delivery, low birth weight, and fetal mortality.^[25]^ Urinary Tract Infections and Kidney Stones (Nephrolithiasis) though not as common as UTIs during pregnancy, Kidney Stones are also a risk factor.^[26]^ Another example of physiological changes that lead to kidney stone formation includes; renal calcium excretion increases coupled with a decrease in urine pH.^[26]^ Nephrolithiasis in pregnancy occurs at a rate of between 1 in 200 to 1 in 1500 pregnancies.^[27]^ Stones may result in renal colic which presents with severe pain and may be interpreted as labor resulting in unnecessary interventions. If not intervened, kidney stones typically lead to obstructive uropathy with a predisposition to infection and preterm labor.^[28]^ Pregnancy has been identified as causing bladder dysfunction that includes urinary incontinence and overactive bladder and up to 50 % of pregnant women suffer from varying degrees of urinary incontinence.^[29]^ The enlargement of the uterus squashes the bladder, thus adding to the urgency statement and the ability to ask for frequent bathroom breaks.^[30]^ These symptoms are worsened by hormonal influences, especially the relaxation of the pelvic floor muscles.^[31]^ Even though bladder dysfunction is not life-threatening, the symptoms can be painful and have a psychological impact especially due to pregnancy burden on the female’s body.^[32]^ Delayed treatment of urological problems during pregnancy has the potential to cause severe consequences on the lives of both the mother and the unborn child as shown in Table 2.^[33]^ Eradicated UTIs may lead to pyelonephritis, which in addition to predisposing women to preterm labor, can lead to life-threatening maternal sepsis.^[34]^ Kidney stones work their way through the urinary system and could cause hydronephrosis and deteriorating renal function; the 2 are dangerous to pregnancy.^[35]^ Urinary bladder dysfunction as compared to fundal height measurement is not life-threatening but it certainly aggravates the quality of life and predisposes to postnatal incontinence.^[29]^ It is, therefore, imperative that these complications are well-managed to achieve positive results.

**Table 1:** Urological complications during pregnancy

Urological complication	Description	Prevalence	Causes/contributors	Potential impact	References
Urinary Tract Infections (UTIs)	Most common urological issue in pregnancy, affecting urinary tract function.	Approximately 10% of pregnant women	Hormonal changes (elevated progesterone), urinary stasis, incomplete bladder emptying. Bacterial growth (primarily *E coli*).	Untreated UTIs can progress to acute pyelonephritis, increasing risks of preterm birth, low birth weight, and fetal mortality.	[4,22,23]
Kidney Stones (Nephrolithiasis)	Less common but significant, causing severe pain and potential complication	Ranges from 1 in 200 to 1 in 1500 pregnancies	Increased renal calcium excretion, decreased urine pH, formation of stones.	Can lead to renal colic, obstructive uropathy, infection, and preterm labor if untreated.	[24–26]
Bladder Dysfunction	Includes urinary incontinence and overactive bladder (OAB).	Up to 50% of pregnant women experience some degree	Pressure from expanding uterus, hormonal relaxation of pelvic floor muscles.	Can cause significant discomfort, emotional distress, and increase risk of postpartum incontinence.	[26–28]

Table 1 shows that common urological complications during pregnancy, such as UTIs, kidney stones, and bladder dysfunction, affect 10% of pregnant women, potentially leading to preterm birth, severe pain, and postpartum issues.

**Table 2:** The impact of urological complications on maternal and fetal health during pregnancy

Urological complication	Potential impact on maternal health	Potential impact on fetal health	References
Urinary Tract Infections (UTIs)	- Can progress to acute pyelonephritis, increasing risk of maternal sepsis. - Increased risk of preterm labor. - Potential for renal damage if untreated.	- Increased risk of preterm birth. - Potential for low birth weight. - Risk of fetal mortality if severe maternal infection occurs.	[29,33]
Kidney Stones (Nephrolithiasis)	Can cause severe pain and renal colic. - Risk of hydronephrosis and impaired renal function. - Potential for obstructive uropathy, which may complicate pregnancy.	Increased risk of preterm labor due to severe pain and complications. - Potential impact on fetal growth if maternal renal function is significantly impaired.	[34,35]
Bladder Dysfunction	Can cause significant discomfort and emotional distress. - Increased risk of postpartum incontinence. - May affect daily functioning and quality of life.	Generally less immediate impact on fetal health. - Possible indirect effects if severe maternal distress affects overall well-being.	[34–36]

Table 2 highlights the maternal and fetal health impacts of urological complications during pregnancy, including UTIs, kidney stones, and bladder dysfunction, which can lead to preterm labor and low birth weight.

### 2.11. Urinary tract infections (UTIs) in pregnancy

Pregnant women are commons carriers of UTIs, one of the most frequent bacterial infections.^[22,28]^ The infections may range from asymptomatic bacteriuria (bacteria in the urine without symptoms) to more serious infections, such as pyelonephritis (kidney infection) that have serious sequelae for both the mother and fetus.^[4,29]^

### 2.12. Complications of UTIs in pregnancy

**Figure 1.**
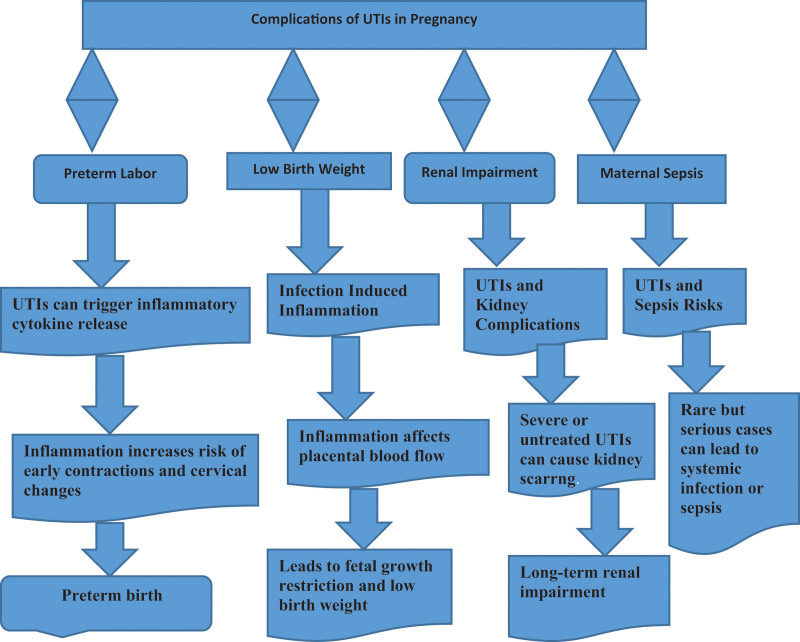
Complications of UTIs in pregnancy. UTIs = urinary tract infections.

In addition, inflammatory cytokines stimulated by UTIs, particularly if the UTIs goes on to cause pyelonephritis, may trigger preterm labor as shown in Figure 1. Inflammation and infection are associated with early contractions and changes in the cervix that may lead to preterm birth.^[23]^ Infection can cause inflammation that can have an adverse effect on placental blood flow and result in fetal growth restriction and low birth weight.^[22]^ The cells involved in the body’s immune response to infection can also affect the growth factors that normally promote fetal development.^[4]^ Untreated severe UTIs especially pyelonephritis, may cause renal complication such as renal scarring leading to long term renal impairment.^[23]^ If the infection spreads to the kidneys it may interfere with renal function by damaging kidney tissue, or in rare but potentially serious circumstances, UTIs may progress to systemic infection (sepsis), which carries serious risks to the mother, including organ failure or death.^[24]^ Sepsis also raises the chance of preterm birth and can shatter the fetal wellbeing.^[23]^

### 2.13. Long term implications of mother and child

Recurrent UTIs or pyelonephritis during pregnancy can lead to chronic kidney problems, including later on in life hypertension and decreased kidney function.^[4,22]^ Even years after pregnancy, these women may be at greater risk for developing renal disease or for developing complications such as chronic hypertension or proteinuria (excess protein in urine).^[23]^ Children born from women who had UTIs during pregnancy are at an increased risk of low birth weight, preterm birth, which increases the risk of neonatal morbidity and mortality.^[24]^ Infants born before term (preterm infants) are at greater risk for respiratory distress syndrome, developmental delays, and long term cognitive and physical impairment. Maternal infection can result in the baby being “small for gestational age” and this may increase the baby’s risk of developing future metabolic or growth problems.^[25,26]^

### 2.14. Complications of kidney stones in pregnancy

Although less common, kidney stones are a possibility during pregnancy, and can be particularly dangerous given the pregnant woman’s changed urinary tract dynamics, hormonal changes, and increased kidney filtration.^[23]^ Kidney stones can cause uterine contractions and can be very painful.^[24]^ Kidney stones themselves may not be the sole cause of labor prepping, but the pain and discomfort as well as the chance of infection all add up to increased risk of preterm birth.^[25]^ Furthermore because of the infection or obstruction, her body may experience stress which could lead to preterm labor if the infection or obstruction is not treated.^[22]^ Enlargement of the renal pelvis (hydronephrosis) during pregnancy, with kidney stones, may cause urine to back up into the kidneys and cause swelling and possible kidney dysfunction. In the worst case scenario this can cause infections, further complications and even permanent kidney damage.^[4]^ Kidney stones which cause a block in the urinary tract can impair kidney function and, if not treated, can cause renal damage.^[23]^ Kidney obstruction in pregnancy is a rare occurrence, and renal failure is a rarely seen, but life threatening, outcome that requires immediate medical intervention.^[24]^ Women who get kidney stones while pregnant may be prone to kidney disease after pregnancy.^[25]^ In particular, chronic kidney disease (CKD) risk may be increased in people who have recurrent stones or damage to the kidneys from obstruction or infection.^[26]^ Women with a history of kidney stones may also be at risk longterm for development of hypertension and cardiovascular disease, in addition to osteoporosis.^[26]^ While kidney stones in pregnancy may not directly impact the child’s health during pregnancy unless complications like infection occur, preterm birth related to kidney stone pain or infection can lead to the same complications as preterm labor from UTIs: At risk for low birth weight, respiratory distress syndrome and long term developmental challenges.^[27]^

### 2.15. The long term implications of chronic kidney disease (CKD) for both mother and child

Recurrent UTIs and kidney stones can each cause kidney damage, including CKD.^[25]^ Affects a woman’s health within the long term, subjecting her to cardiovascular disease, hypertension and proteinuria.^[23]^ Some of these problems will need monitoring, they may require changes in lifestyle and in some cases medication to keep control of kidney health.^[26]^ For example, women who have kidney problems before pregnancy, or who develop renal impairment during pregnancy, may accelerate the decline in their kidney function as they age.^[25]^ Women who have chronic kidney issues or recurrent infections can experience a greatly diminished quality of life from pain, fatigue and the psychological cost of living with a chronic illness.^[23]^ Emotional wellbeing, relationships, physical health may also be affected by this. Renal infection or the stress associated with a kidney stone can lead to preterm birth and intrauterine growth restriction (IUGR) causing lifelong neurodevelopmental problems in a child.^[24]^ In particular, low birth weight babies face a greater risk for developing a delay in development, such as learning disability, or behavioral challenge.^[25]^ Having been born preterm or with low birth weight may increase the chances that children will develop chronic health conditions in later life, including asthma, cardiovascular disease and diabetes.^[26]^ These conditions put people at an increased risk, because they experienced the stress as it occurred during fetal development and infancy. UTIs and kidney stones are common complications in pregnancy, but neglect the potential they have for causing major, lasting health problems.^[26]^ With both conditions one is predisposed to develop preterm labor, low birth weight and renal dysfunction, and the consequences of which may extend into the life of the mother and the child.^[27]^ If these complications are addressed early and carefully managed during pregnancy, risks can be minimized and the best possible outcomes for both mother and baby can be achieved.^[28–31]^ This requires early diagnosis, adequate treatment and continuing observation to minimize long term effects on maternal and child health.^[32–35]^

### 2.16. Pharmacological management of urinary tract infections (UTIs) in pregnant women

Pregnant women are prone to UTIs as the renal pelvis and ureters dilate, and the lower urinary tract progressively relaxes as pregnancy advances, leading to urinary stasis as in Table 3.^[36]^ The pharmacological intervention of UTI in pregnancy is important to avoid the extension of the infection to Severe forms like acute pyelonephritis which affects fertility and fetal development.^[37]^ Antibiotics are the mainstay of treatment for UTIs in pregnancy, but the selection of antibiotics should be balanced both by their efficacy and potential toxicity for the fetus.^[38]^ Most first-line antibiotics are beta-lactams including amoxicillin, ampicillin, and cephalexin; and these drugs have been considered safe in pregnancy.^[24]^ Cephalexin is often used because it is a first-generation cephalosporin with a wide range of activity against uropathogens and is not likely to pose a teratogenic risk.^[39]^ Nitrofurantoin is another widely used antibiotic particularly for the first-line treatment of UTIs, although it should be prescribed within the last trimester because it can cause neonatal hemolysis.^[40]^ Antibiotics can be administered to the pregnant woman only if it is justified by the nature and severity of the disease, in order not to harm the fetus. For example, nitrofurantoin is very effective against Gram-negative bacteria; however, there is evidence that its use in the third trimester can be dangerous because it can cause hemolysis in neonates with G6PD deficiency.^[41,42]^ On the other hand, fluoroquinolones and tetracyclines cannot be prescribed to pregnant women since these drugs can affect fetal cartilage and lead to tooth discoloration respectively.^[43]^ Based on these risks, antibiotic therapy should consider the results of culture and sensitivity as a way of ensuring that the selected antibiotic will deal with the pathogen.^[44]^ It is essential to note that UTIs during pregnancy require longer antibiotic courses compared to non-pregnant women due to the physiological changes in pregnancy and its physiological effects, typically to 7 to 14 days depending on the severity of the infection.^[24]^ Shorter courses may not accomplish the complete obliteration of infection and as a result, make a cycle reoccurring. Further urine cultures are suggested to ensure bacteriological cure because Asymptomatic Bacteriuria is frequent during pregnancy and its mishandling may result in complications.^[45]^ Regarding recurrent UTIs, the patient may be prescribed antibiotics for preventive purposes to minimize the risk of getting a UTI in the future. Another of the emergent complications of UTIs during pregnancy is the constant rise in the rates of antibiotic-resistant bacteria especially multidrug-resistant E. coli.^[46]^ This has encouraged the use of culture and sensitivity tests to determine the appropriate antibiotics to use. Empiric therapy should be discouraged in locations where rates of resistance are elevated, as well as using narrow-spectrum antibiotics in preference to broad-spectrum agents to limit the emergence of resistance.^[47]^ It is important therefore for women with a history of recurrent UTIs or other risk factors like diabetes or a weakened immune system to be adequately monitored as they are pregnant as they are at a higher risk of complications of the UTIs. Sustainable urine examinations and subsequent evaluations are mandatory to corroborate the complete eradication of the infection as well as to reveal possible dramatications of the process, including pyelonephritis or preterm labor. Furthermore, there is a need to ensure patient enlightenment on issues to do with water intake, regular washing, bathing, and compliance with the prescribed doses of antibiotics required to eradicate the relapse.^[48]^

**Table 3:** The pharmacological management of urinary tract infections (UTIs) in pregnant women

Aspect	Details	References
First-Line Antibiotics Safe for Use During Pregnancy	- **Amoxicillin**: Effective against common uropathogens, well-tolerated. - **Ampicillin**: Broad-spectrum, safe in pregnancy. - **Cephalexin**: First-generation cephalosporin, broad-spectrum, minimal teratogenic risk. - **Nitrofurantoin**: Effective for uncomplicated UTIs, avoid in the last trimester due to risk of neonatal hemolysis.	[36–38]
Risks and Benefits of Different Antibiotics	**Nitrofurantoin**: Effective but may cause hemolytic anemia in neonates with G6PD deficiency; generally avoided in the third trimester. - **Fluoroquinolones**: Contraindicated due to potential fetal cartilage damage. - **Tetracyclines**: Contraindicated due to potential tooth discoloration.	[39–42]
Guidelines for Treatment Duration and Follow-Up	**Treatment Duration**: Typically 7 to 14 days; longer courses are preferred to ensure complete eradication. - **Follow-Up**: Repeat urine cultures recommended to confirm bacterial clearance and manage asymptomatic bacteriuria (ASB). - **Recurrent UTIs**: Consider prophylactic antibiotics.	[43–45]
Managing Antibiotic Resistance	**Resistance Issues**: Increasing prevalence of multidrug-resistant E. coli. - **Culture and Sensitivity Testing**: Essential for selecting effective antibiotics. - **Empiric Therapy**: Should be avoided in areas with high resistance rates; prefer narrow-spectrum antibiotics.	[44–46]
Monitoring and Follow-Up Care	**Close Monitoring**: Essential for those with recurrent infections or higher risk (e.g., diabetes, immune compromise). - **Regular Urine Cultures**: Important to ensure infection resolution. - **Patient Education**: Emphasize hydration, proper hygiene, and completing the antibiotic course.	[47–49]

Table 3 outlines pharmacological management of UTIs in pregnant women, including first-line antibiotics, follow-up urine cultures, antibiotic resistance testing, and patient education on hydration and hygiene.

### 2.17. Management of acute pyelonephritis in pregnancy

Acute pyelonephritis is one of the most severe complications of untreated UTIs during pregnancy, ranging from 1% to 2% of pregnant women as shown in Table 4.^[49]^ It is accompanied by increased maternal and fetal morbidity such as preterm labor, sepsis, and low birth weight. However, these adverse outcomes should receive prompt and adequate treatment to be prevented.^[50]^ Acute pyelonephritis during pregnancy should be treated in the hospital, with more intense antimicrobial therapy if there is a sign of systemic infection or high temperature. Emergency treatment usually involves starting with intravenous (IV) antibiotics like ceftriaxone, cefazolin ampicillin, and gentamicin.^[49]^ These antibiotics are chosen because of their spetrogram, which proves their low teratogenic effect, and effectiveness against the most frequent uropathogenic microorganisms, such as Escherichia coli. If the patient is apyrexial, clinically well, and has no signs of infection after 48 hours the intravenous antibiotics can be switched to oral antibiotics such as amoxicillin/clavulanic acid, or cephalexin, for a total duration of 10 to 14 days of antibiotics.^[49]^ Although most cases of acute pyelonephritis in pregnancy do require hospitalization, IV antibiotics, and monitoring, some mild cases may be treated on an outpatient basis. Outpatient management is usually indicated in patients who have no other signs of systemic illness, no history of recurrent UTI, and are unable to take oral medicines.^[49]^ However, this is not often usually the case because pyelonephritis demands IV treatment to effectively clear out the bacteria and to avoid the subsequent formation of sepsis, ARDS, or preterm labor.^[34]^ Besides antibiotic treatment, strict surveillance for the development of complications like ARDS, septic shock, and preterm labor must be done. ARDS is a lethal complication that develops in roughly 2% of pregnant women with acute pyelonephritis and should be treated in an intensive care setting.^[34]^ Others include the administration of IV fluids and antipyretics to address fevers since patients should be encouraged to produce urine. Furthermore, obstetric surveillance is necessary to look for evidence of preterm labor, which is a relatively frequent occurrence in women with pyelonephritis. In some cases, treating the mother with corticosteroids to accelerate the development of the lungs of the unborn baby can be required if pre-term labor is expected.^[51]^ In the management of acute pyelonephritis in pregnancy, therefore, the aim should be to rapidly treat the infection, reduce the likelihood and severity of complications, and safeguard the health of both the mother and the fetus.^[52]^

**Table 4:** Management of acute pyelonephritis during pregnancy

Aspect	Details	References
Recommended Antibiotic Regimens for Severe UTIs	**Initial Treatment**: Intravenous (IV) antibiotics such as ceftriaxone, cefazolin, or ampicillin combined with gentamicin. - **Transition to Oral Therapy**: Once afebrile for 48 hours and clinically stable, switch to oral antibiotics like amoxicillin-clavulanate or cephalexin. - **Duration**: Total antibiotic course typically lasts 10 to 14 days.	[49–51]
Hospitalization Versus Outpatient Management	**Hospitalization**: Generally required for IV antibiotic therapy and close monitoring of systemic symptoms, high fever, and potential complications. - **Outpatient Management**: Rare; reserved for mild cases without systemic symptoms, no history of recurrent UTIs, and ability to tolerate oral antibiotics.	[34,49]
Monitoring and Managing Potential Complications	**Complications**: Monitor for acute respiratory distress syndrome (ARDS), septic shock, and preterm labor. - **ARDS**: Occurs in about 2% of cases; requires intensive care. - **Septic Shock**: Requires immediate management and intensive monitoring. - **Preterm Labor**: Regular obstetric monitoring; administer corticosteroids if preterm birth is imminent. - **Supportive Care**: IV hydration and antipyretics to reduce fever and maintain urine output.	[51,52]

Table 4 outlines pregnancy-specific management of acute pyelonephritis, including IV antibiotics, hospitalization, outpatient care, and monitoring for complications like ARDS, septic shock, and preterm labor.

### 2.18. Treatment of kidney stones during pregnancy

Pregnancy-associated nephrolithiasis is a rare occurrence, affecting roughly 1 in 1500 pregnant women as shown in Table 5.^[53]^ However, there are always potential complications such as extreme pain, risk of infection, and preterm labor. pretreatment of kidney stones during pregnancy is difficult since the treatment involves balancing the health risks between mother and child.^[54–56]^ Conservative treatment is usually the rule and medications are limited to analgesics and antibiotics as necessary.^[56–59,60]^

**Table 5:** The treatment of kidney stones during pregnancy

Category	Treatment options	Considerations	References
Incidence	Rare (1 in 1500 pregnancies)	Kidney stones can cause severe pain, infection, and preterm labor	[53–55]
Pharmacological Management	Acetaminophen	First-line analgesic; safe throughout pregnancy	[56]
	Opioids (e.g., Morphine, Hydrocodone)	Used for severe pain; requires strict medical supervision due to risks of sedation and neonatal withdrawal	[57]
	NSAIDs (e.g., Ibuprofen)	Generally avoided after the first trimester due to risks of fetal renal impairment and premature ductus arteriosus closure.	[58,59]
Alpha-Blockers	Tamsulosin	Used to facilitate stone passage by relaxing ureters; reserved for significant discomfort or obstruction.	[56]
Calcium Channel Blockers	Nifedipine	Similar use as alpha-blockers; consider risk-benefit profile in low doses under medical supervision.	[^57,60^]
Non-Pharmacological Treatments	Ureteral Stenting	Preferred for relieving obstruction; can be done under local or regional anesthesia with minimal fetal risk.	[61]
	Percutaneous Nephrostomy	Another option for relieving obstruction; minimal fetal risk.	[62]
	Ureteroscopy	Minimally invasive; used when other interventions fail.	[63]
Surgical Options	Extracorporeal Shock Wave Lithotripsy (ESWL)	Contraindicated during pregnancy due to potential fetal injury.	[63]

Table 5 outlines pregnancy treatment options for kidney stones, including acetaminophen, opioids, NSAIDs, tamsulosin, nifedipine, non-pharmacological treatments, and ESWL due to potential fetal harm.

### 2.19. Bladder dysfunction in pregnancy

About 50% of pregnant women suffer from bladder dysfunction during pregnancy.^[61–64]^ Usually, these problems stem from the physical changes of pregnancy such as hormone changes, extra blood volume, and a urinary bladder and pelvic structures squeezed by the growing uterus.^[65]^ Bladder dysfunction can be temporary after delivery, but it can also affect a woman’s quality of life physically and emotionally.^[66]^

### 2.20. types of bladder dysfunction in pregnancy

Urinary incontinence and urinary retention are the 2 most often seen forms of bladder dysfunction in pregnancy.^[67]^ However, the 2 conditions are related in that both stem from effects that pregnancy has on bladder function, but present themselves differently and are managed differently.^[68]^

### 2.21. Urinary incontinence

**Figure 2.**
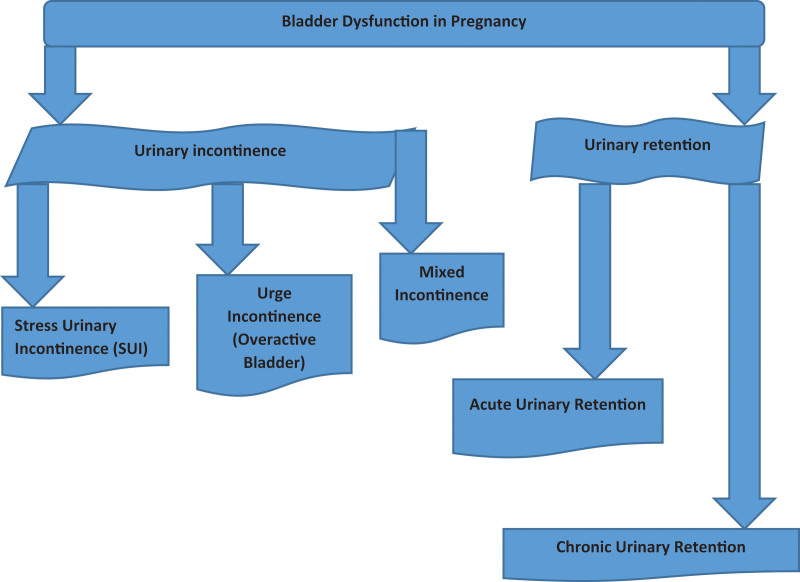
Types of bladder dysfunction in pregnancy.

Urinary incontinence is a bladder dysfunction characterized by involuntary leakage of urine and is probably the most common form of bladder dysfunction seen during pregnancy as shown in Figure 2. It can manifest as:

Stress Urinary Incontinence (SUI): The most common type of urinary incontinence in pregnant women is this. This happens with increased intra-abdominal pressure, with coughing, sneezing, laughing, or exercise.^[66,67]^ Pregnancy causes your uterus to grow and put pressure on your bladder, and hormonal changes and the physical demands of pregnancy can make your pelvic floor muscles become weak or overstretched. Urine leakage occurs during activity that increases abdominal pressure.^[67]^

Urge Incontinence (Overactive Bladder): The type in which there is a strong, urgent need to urinate that also may be involuntary loss of urine.^[65]^ Hormones taking over as progesterone increases cause the bladder muscles to contract too frequently or unpredictably.^[64]^ Urgency and frequency of urination can also be aggravated as the pregnancy’s growing uterus irritates the bladder.^[66]^

Mixed Incontinence: Other women have a combination of stress and urge incontinence, a condition known as mixed incontinence.^[67]^ This often happens during pregnancy, and most particularly in the later stages, during which the pelvic floor muscles may become weakened, and the bladder overactive.^[68]^

### 2.22. Urinary retention

Urinary retention is the inability to empty all of the urine from the bladder or having trouble getting the urine stream started.^[68]^ Although it is less common during pregnancy the condition does occur, most often when the pelvic muscles are weaker or the growing uterus places pressure on the urinary tract.^[69]^ Acute Urinary Retention: It means you cannot suddenly urinate, which is a medical emergency.^[70]^ The cause may simply be severe pressure on your bladder or urethra, an issue you may experience in the later stages of pregnancy when the baby falls into a position that blocks the normal flow of urine.^[71]^ Urinary retention can also be caused by hormonal changes (essentially the effects of progesterone) in pregnancy, which relax the muscles of the bladder and urethra and impact normal bladder function.^[72]^

Chronic Urinary Retention: It causes you to feel like you have not finished emptying your bladder and that you are constantly going to the bathroom.^[71]^ If left untreated over time, this can lead to urinary tract infections (UTIs) or even kidney problems.^[72]^

### 2.23. Impact of bladder dysfunction on quality of life

Pregnancy bladder dysfunction can significantly affect a woman’s level of life quality.^[65]^ The symptoms of incontinence and retention can interfere with daily activities, work, and social interactions, leading to:

Physical Discomfort: The woman might be rushing to the bathroom more often than usual, often having a sense of urgency in addition to a fear of leakage causing physical discomfort.^[65]^ Stress incontinence can hinder exercise, work around the house or just casual everyday activities.^[66]^

Emotional and Psychological Effects: Women with bladder dysfunction may feel embarrassed, anxious, frustrated about bladder dysfunction.^[67]^ Urinary leakage may cause them to feel self conscious or may keep them from being social, which can result in isolation or not feeling good about themselves.^[65]^ In others, depression or low self esteem may become issues for women with the physical limitations or a loss of control over the bodies.^[68]^

Impact on Sleep: Another symptom of frequent urination during pregnancy is nocturia, which can disrupt your sleep extremely.^[65]^ Frequent awakenings to use the bathroom can make you fatigued, irritable and unable to concentrate during the day.

Sexual Health: A woman’s sexual health can be affected by stress urinary incontinence or urinary retention.^[64]^ Urinary distress resulting from an inability to inhibit the urethral sphincter when urinating may make sexual activity distressing while bladder retention may lead to discomfort or decreased sexual desire.^[67]^

### 2.24. Management of bladder dysfunction in pregnancy

**Figure 3.**
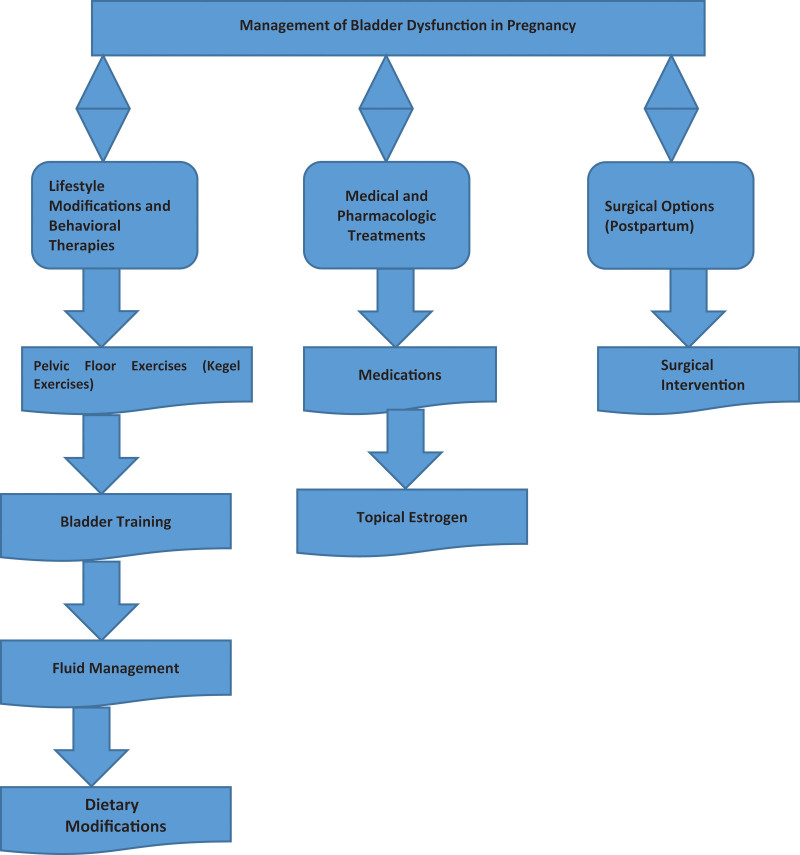
Management of bladder dysfunction in pregnancy.

Bladder dysfunction management during pregnancy consists of a variety of lifestyle changes, physical therapy and medical interventions as needed as shown in Figure 3. There are few cures for the conditions listed below and most treatment approaches are designed to relieve symptoms and improve quality of life.^[64]^

### 2.25. Lifestyle modifications and behavioral therapies

Pelvic Floor Exercises (Kegel Exercises): One of the best treatments for stress urinary incontinence in pregnancy is doing Kegel exercises.^[64–66]^ To stretch the pelvic floor muscles, which help support the bladder, uterus and bowel, these help.^[65]^ Regular practice can decrease the amount that leaks out of the bladder, help you regain control of your bladder and relieve symptoms of incontinence. Postpartum, it’s also possible to prevent urinary incontinence by doing pelvic floor exercises.^[66]^

Bladder Training: Bladder training involves training your bladder to hold pee longer.^[65]^ This can be especially useful for women with urge incontinence or have to urinate a lot.^[67]^ These are attempts to retrain the bladder to “hold” more urine for longer periods.^[66]^

Fluid Management: Although pregnancy increases women’s need for hydration, limiting fluid intake in the hours before bedtime may help to minimize nocturia (nighttime urination). Stopping caffeinated beverages may also help, as they can irritate the bladder.^[64]^

Dietary Modifications: For women who experience bladder symptoms, avoiding foods and drinks that bother the bladder, like acidic foods and spicy foods, citrus fruits, and artificial sweeteners, may help them feel better.^[68]^

### 2.26. Medical and pharmacologic treatments

Medication: For really bad cases of urge incontinence, medications called anticholinergics (e.g. oxybutynin), which relax the bladder and reduce urgency, may be prescribed.^[69,70]^ Most of these medications are not used in pregnancy because of side effects and are considered only after non pharmacologic treatment has failed.^[71]^

Topical Estrogen: Low dose topical estrogen can even be prescribed in some cases to strengthen vaginal and pelvic floor muscles, in women with history of menopause or post menopausal symptoms.^[72]^

### 2.27. Surgical options (postpartum)

Surgical Intervention: If conservative treatments are unsuccessful in relieving persistent bladder problems experienced by women after pregnancy, surgery is considered.^[57]^ These surgeries (sling procedures or other surgeries) aren’t options to correct pelvic organ prolapse or incontinence.^[66]^ Usually these options are considered after the postpartum period when the body heals and gets back to its normal state.^[68]^

### 2.28. Prevention strategies for bladder dysfunction

While it may not be possible to prevent bladder dysfunction entirely, there are several strategies that can reduce the risk or minimize the severity of symptoms:

Regular Pelvic Floor Exercises: Doing pelvic floor exercises from early in your pregnancy and continuing afterwards helps tone the muscles around the bladder, which make incontinence less likely.^[67]^

Maintaining a Healthy Weight: If you are carrying excess weight, particularly in later pregnancy, this pressure can exert itself on your bladder and pelvic floor muscles, causing leakage.^[68]^ Enjoying an overall healthy weight by keeping a balanced diet and doing a moderate exercise will also help relieve the pressure on the bladder.^[69]^

Avoiding Constipation: Bladder dysfunction is common during pregnancy and can be made worse with constipation.^[71]^ Constipation stemming from a high fiber diet, adequate hydration, and regular physical activity can help prevent constipation and go the distance in reducing pressure on the bladder.^[72]^

Education and Awareness: If women are educated early about bladder health and potential dysfunction, they will know the signs early and how early to get treatment.^[73]^ By taking a proactive approach, emotional burden of bladder dysfunction can be reduced and overall outcomes improved.^[74]^

Urinary dysfunctions such as urinary incontinence, and urinary retention are common during pregnancy and can strongly disturb quality of life in women.^[64]^ These conditions are quite frequent, but only occasional, i.e. they normally pass over time but will result in discomfort, emotional distress and physical limitations.^[68]^ Usually effective management includes a combination of lifestyle changes, pelvic floor exercises, behavioral therapies and in some cases medication.^[69]^ Early diagnosis of these problems can decrease symptoms and improve quality of life for women during pregnancy and beyond.^[70]^ After childbirth, recurrence of bladder dysfunction can be prevented by using strategies on longer term: regular pelvic floor exercises and healthy lifestyle choices.^[71]^

### 2.29. Pharmacological options for pain management

Pain that results from kidney stones can be severe, and in such cases, there may be the need to use drugs. However, the use of analgesics in pregnancy is limited because of potential fetal risk.^[56]^ NSAIDs like Ibuprofen are contraindicated especially from the second trimester because it has been linked to impaired fetal renal function and premature shutdown of ductus arteriosus.^[57]^ However, the drug of choice for analgesia is often acetaminophen because it is said to be safe during pregnancy.^[58]^ For severe pain in pregnancy, opioids like morphine or hydrocodone may be administered but with extreme caution because of the effects that the drugs may have on the pregnant women which include maternal sedation and possibilities of neonatal withdrawal.^[59]^

### 2.30. Safe use of medications like alpha-blockers and calcium channel blockers

Pain that results from kidney stones can be severe, and in such cases, there may be the need to use drugs. However, the use of analgesics in pregnancy is limited because of potential fetal risk.^[56]^ NSAIDs like Ibuprofen are contraindicated especially from the second trimester because it has been linked to impaired fetal renal function and premature shutdown of ductus arteriosus.^[57]^ However, the drug of choice for analgesia is often acetaminophen because it is said to be safe during pregnancy. For severe pain in pregnancy, opioids like morphine or hydrocodone may be administered but with extreme caution because of the effects that the drugs may have on the pregnant women which include maternal sedation and possibilities of neonatal withdrawal.^[60]^

### 2.31. Non-pharmacological treatments and surgical options

Alpha blockers such as tamsulosin and calcium channel blockers like nifedipine have been applied to promote the expulsion of kidney stones through the ureters since they are comprised of smooth muscles.^[61]^ Despite scarce literature available regarding the safety of these medicines during pregnancy, these medications have been reported to have a favorable risk/benefit ratio especially when taken in small doses and under the doctor’s prescription.^[62]^ Alpha-blockers as a rule are employed in cases when nonsurgical intervention has not resolved issues that make the patient uncomfortable or experiencing constipation and obstruction.^[63]^

### 2.32. Pharmacological approaches to managing bladder dysfunction

Bladder dysfunction, including urinary incontinence and OAB, affects a significant proportion of pregnant women as shown in Table 6.^[64]^ It is estimated that between 30% and 50% of women experience some form of urinary incontinence during pregnancy.^[29]^ The growing uterus places pressure on the bladder, and hormonal changes relax the pelvic muscles, exacerbating these symptoms. Managing these conditions effectively is crucial to improving the quality of life for pregnant women.^[65]^

**Table 6:** Pharmacological approaches to managing bladder dysfunction in pregnant women, focusing on the safety profiles of various medications

Approach	Medication	Mechanism of action	Safety profile in pregnancy	Comments	References
First-line Medications for Overactive Bladder (OAB)	Oxybutynin	Antimuscarinic; inhibits bladder activity	Generally discouraged; limited safety data available	Can cross placental barrier; potential teratogenic risks	[66]
	Solifenacin	Antimuscarinic; inhibits bladder activity	Generally discouraged; limited safety data available	Can cross placental barrier; potential teratogenic risks	[67]
	Tolterodine	Antimuscarinic; inhibits bladder activity	Generally discouraged; limited safety data available	Can cross placental barrier; potential teratogenic risks	[68]
Non-Pharmacological Approaches	Pelvic Floor Muscle Training (PFMT)	Strengthens pelvic muscles; improves bladder control	Safe and recommended	Effective for managing OAB and urinary incontinence during pregnancy	[29]

Table 6 discusses pharmacological and non-pharmacological methods for managing bladder dysfunction in pregnant women, emphasizing non-pharmacological methods like pelvic floor muscle training as safe and recommended.

### 2.33. Medications for overactive bladder and their safety profiles

OAB represents the urge urinary incontinence that arises due to the involuntary contraction of the detrusor muscle.^[66]^ Oxybutynin, solifenacin, and tolterodine are examples of antimuscarinic medications that are used for patients with OAB but not pregnant ones.^[67]^ Nonetheless, their take during pregnancy is considered unsafe because of the lack of data regarding their safety profiles and any possible teratogenic actions they may pose. Antimuscarinic drugs block the muscarinic receptors in the bladder thereby decreasing detrusor overactivity, yet they may cross the placental barrier thereby influencing fetal development.^[68]^ Due to the lack of research regarding the safety of drugs for OAB in pregnant women any pharmacological treatments are considered only if the condition the woman experiences is significantly affecting her quality of life.^[69]^ Hence, non-drug intervention; pelvic floor muscle training (PFMT) should be encouraged as research also shows that PFMT can help manage the symptoms of OAB and UI among pregnant women.^[29]^

### 2.34. Management of urinary incontinence during pregnancy

Stress urinary incontinence (SUI) is the predominant type of incontinence experienced during pregnancy due to raised intra-abdominal pressure acting on the bladder as shown in Table 7.^[70]^ Anti-muscle inactivating agents, including duloxetine, are also contra-indicated in pregnant women due to the dangers that the fetus may suffer.^[71]^ Duloxetine, a serotonin-norepinephrine reuptake inhibitor, elevates the tone of the urethral sphincter muscles but can cause miscarriages and birth defects.^[72]^ While practicing pharmacological approaches for the treatment of SUI, pregnancy-related SUI is mainly treated by behavioral therapies like bladder training and PFMT. These conservative therapies focus on making the pelvic floor muscles and the bladder wall stronger to minimize the degree of incontinence.^[29]^ In the case of the surgery, during pregnancy, operations are not carried out because of the possible consequences and are done after shedding the baby.^[73]^

**Table 7:** The management of urinary incontinence during pregnancy, focusing on stress urinary incontinence (SUI) and alternative treatments

Management approach	Details	Effectiveness	Comments	References
Pharmacological Treatments	Duloxetine	Not recommended during pregnancy	Associated with increased miscarriage and birth defects	[72]
Behavioral Therapies	Pelvic Floor Muscle Training (PFMT)	Highly effective for prevention and treatment	Recommended for both pregnancy and postpartum	[49]
	Bladder Training	Effective for OAB and urinary incontinence	Involves scheduled voiding to improve bladder capacity	[71]
Surgical Interventions	Typically deferred until after childbirth	Rarely performed during pregnancy	Risks of complications make it impractical during pregnancy	[72]
Alternative Treatments	Electrical Stimulation and Biofeedback	Safety and efficacy during pregnancy uncertain	Not typically recommended unless under medical supervision	[71]

Table 7 discusses pregnancy urinary incontinence management, emphasizing behavioral therapies like pelvic floor muscle training and bladder training, with surgical interventions deferred due to risks.

### 2.35. Alternative treatments and their effectiveness

Since the pharmacological treatments for bladder dysfunction in pregnancy are marginal, much reliance on their control emanates from non-pharmacological remedies.^[74]^ The PFMT, which is also called Kegel exercises, is a very effective preventive measure as well as utilized for managing UI during and after pregnancy. Several systematic reviews have revealed that PFMT reduces the risk of postpartum incontinence among women who practice it regularly during pregnancy based on sample randomized controlled trials.^[75]^ Bladder training which is a formularized schedule of voiding and timed interventions to prolong the time between bathroom trips is also beneficial in treating OAB and incontinent patients in the long run.^[76]^ However, while electrical stimulation and biofeedback are employed in non-pregnant patients suffering from bladder dysfunction, their safety and effectiveness during pregnancy have not been established, and thus they should not be employed during pregnancy except by prescription from a doctor.^[77]^

### 2.36. Impact of pharmacological interventions on fetal development

The use of pharmacological intervention during pregnancy has often been associated with detrimental effects to the offspring as shown in Table 8.^[78]^ These are some of the main issues: teratogenicity, fetal toxicity, and the influence of a drug on the further development of a fetus. It remains to consider which medications are safer for mother and child when the woman herself suffers from hypocomplacentinemia, has vesical pain, or has complications like zonal necrosis, gestational diabetes, diabetic retinopathy, or endometrial cancer.^[79]^

**Table 8:** The impact of pharmacological interventions on fetal development, focusing on teratogenic risks, potential long-term effects, and monitoring strategies

Aspect	Details	Examples	Considerations	References
Teratogenic Risks	Medications can cause developmental malformations, especially during the first trimester.	Fluoroquinolones (cartilage damage)	Contraindicated in pregnancy	[78,79]
		Tetracyclines (tooth discoloration, bone growth inhibition)	Contraindicated in pregnancy	[80]
Medications with Minimal Teratogenic Risk	Some antibiotics are considered safe and effective during pregnancy.	Beta-lactam antibiotics (e.g., penicillins, cephalosporins)	Minimal teratogenic risk (Angelescu et al., 2016)	[81]
Long-Term Developmental Effects	Medications may lead to long-term consequences that are not immediately evident.	Opioids (neonatal abstinence syndrome)	Monitor for withdrawal symptoms in newborns (Jolley & Wing, 2014)	[82]
			Antimuscarinic drugs (potential neurodevelopmental effects) Limited data in	[83]
Fetal Programming	Environmental factors, including maternal medication use, can influence long-term health outcomes.	Possible increased risk of chronic conditions (asthma, obesity, cardiovascular disease)	Ongoing research needed (Foxman, 2014)	[84,85]
Monitoring Strategies	Routine monitoring of fetal development is essential when medications are necessary.	Routine ultrasounds	Detect abnormalities in growth and development	[86]
		Non-stress tests and biophysical profiles	Assess fetal well-being if risks are known (Cunningham & Lucas, 1994)	
		Fetal echocardiography for congenital heart defects	Recommended for mothers on certain medications	

Table 8 highlights pharmacological interventions’ impact on fetal development, highlighting teratogenic risks, long-term effects, and monitoring strategies. Environmental factors and medication use affect long-term health outcomes.

### 2.37. Assessing the teratogenic risks of commonly used medications

Teratogenicity deals with the capacity of a substance to produce developmental abnormalities in the fetus usually in the first trimester of gestation period when organ formation begins.^[87]^ Some drugs are known to cause teratogenic effects and therefore should not be given during pregnancy including fluoroquinolones and tetracycline.^[88]^ This is of course next to Fluoroquinolones which are known to cause cartilage damage as well as alter the conformation of bone proteins making them brittle to the point of breaking; Tetracyclines on the other hand are known to cause tooth discoloration and inhibit bone growth in the fetus.^[80]^ Beta-lactam antibiotics like penicillin and cephalosporin are considered safe in pregnancy and are often used in managing UTI and pyelonephritis.^[81]^ These antibiotics have been reported to have low teratogenic effects yet they are efficient bacterial eradication and treatment without affecting the fetus in a very adverse manner.^[89]^ Hence, unlike other medications, all drugs including the preferably safe ones should be taken only when necessary, especially during the first trimester of pregnancy.^[90]^

### 2.38. Evaluating potential long-term effects on the fetus

However, one needs to go a step further and also look at the long-term teratogenic effects of the medications developed. Short-term use may not have reversible adverse effects on the developing fetus, far reaching effects of certain drugs may manifest themselves over time.^[82]^ For instance, studies show that the use of opioids in the management of pain during pregnancy leads to neonatal abstinence syndrome (NAS) which results in withdrawal in babies after birth.^[83]^ Likewise, antimuscarinic drugs used in OAB treatment possess neurodevelopmental interactions, although, the pregnant population’s science is insufficient. Pregnancy programming or the idea that maternal health, diet, and medications taken during pregnancy can impact the child’s future health are among the emerging fields of research.^[91]^ Despite the current ambiguity of the relationship, several studies recommend that maternal medication use during pregnancy can predict the incidence of chronic diseases like asthma, obesity, and cardiovascular disease later in life.^[92]^ This helps to put into perspective the need to exercise a lot of caution when choosing medication during pregnancy.

### 2.39. Monitoring fetal development during treatment

In cases when it is decided that medications are required during pregnancy, close supervision of fetal growth is imperative.^[84]^ Ultrasound scans are carried out at regular intervals, which allows identifying such developmental issues with the fetus as may be caused by medication taken by the mother. In cases when the woman takes medications with risks, like opioids or antimuscarins, extramural tests, non-stress tests, or biophysical profiles to evaluate the fetus may be further necessary.^[85]^ At times a fetal echocardiogram may be employed for screening if the mother has taken medications that potentially induced congenital cardiac disorders.^[86]^ Both maternal and fetal health should always be considered and management of pregnant women should always involve discussions between obstetricians, urologists, and pharmacologists.^[84]^

### 2.40. Drug safety and guidelines for pharmacological use during pregnancy

Medication management in pregnancy always takes into consideration risks to the mother as well as risks to the unborn child.^[93]^ Drug safety is evaluated according to the possible teratogenicity of drugs during pregnancy, their toxicity for the fetus, and their effect on further fetal development. Several associations including ACOG and the FDA have issued protocols and drug categorization to assist in the decision making process.^[94]^

### 2.41. Category classifications of drugs and their implications

The Pregnancy and Lactation Labeling Rule (PLLR), which came into effect in 2015, changed the system of Risk Categories (A, B, C, D, and X) to contain specific information on the safety of medication during pregnancy and lactation.^[95]^ About pregnancy, the new labeling format contains details on risk information associated with pregnancy, fetal development, and effects during lactation. This approach is useful in determining the overall risk for the use of medication during pregnancy as distinguished by human and animal data to give a better picture of safety.^[96]^ Before this change, Category A was acknowledged as being safe for pregnancy since these drugs had not shown any adverse effects on the fetus when the pregnancy was controlled. Category B drugs were also considered to have a low risk of causing birth complications; there were no controlled studies conducted on animals revealing any potential damages to the fetal organ, or; in the cases of human medical investigations, no proper controlled studies revealing the risks of the drug intake during pregnancy.^[97]^ Category C drugs have some effects on animals, but the medicinal use by pregnant women may sometimes be preferred due to the many benefits that come with it. Category D was associated with risks that were characterized as obvious, and Category X was contraindicated due to risks that were confirmed through teratogenicity, such as those observed in isotretinoin.^[98]^

### 2.42. Recommendations from professional organizations

ACOG and the CDC are indispensable sources for guidelines regarding pharmacological treatments during pregnancy.^[99]^ According to ACOG, it is essential to use medications with safety data during pregnancy, especially because first-trimester exposure is associated with the highest risk of teratogenicity.^[100]^ Penicillin, amoxicillin, and cephalexin are commonly prescribed for UTIs during pregnancy because they are unlikely to harm the fetus. The CDC also offers tips on how pregnant women should be managed in diseases such as uncomplicated urinary tract infections, acute pyelonephritis, and renal calculi. Possibly unsafe drugs, including fluoroquinolones and tetracyclines, should not be used by pregnant women.^[101]^ The options for the management of pain include acetaminophen which is advocated for unlike NSAIDs given their risks towards the later gestational period.^[102]^

### 2.43. Personalized medicine approaches and patient-specific considerations

Drug and pregnant woman interactions require individual attention more often than not. Secondary effects of therapies should not be also excluded and other aspects that are connected with each patient and Pregnancy weeks.^[103]^ For example, women who could present with a past history of recurrent UTIs, or asymptomatic bacteriuria, should then be considered for long-term antibiotic prescription depending on their risk factors, and the antibiotic resistance of the particular bacteria in concern.^[104]^ The utility of the treatment requires demonstrating a high level of concern while controlling and monitoring the mother and the fetus during its delivery. As for the pharmacological intervention to treat cardiac dysrhythmias, the clinicians are informed that they act as needed under the circumstances.^[105]^ Therefore, when the risk of morbidity of associated maternal disease is greater than the risk of the said drugs, which may cause different complications in the course of further treatment, severe diseases including pyelonephritis or obstructive nephropathy due to renal stones, drugs that should be avoided must be used.^[103]^

### 2.44. Case studies and clinical trials on pharmacological management of urological issues

Recent research and clinical trials offer valuable insights into the safety and efficacy of pharmacological treatments for managing urological complications during pregnancy. Evidence-based practices from these studies help guide clinical decisions and improve maternal and fetal outcomes.^[106]^

### 2.45. Review of recent research and clinical trials

Wing et al^[49]^ performed one of the biggest quantitative research on acute pyelonephritis in pregnancy analyzing more than a decade of hospital archives. The researcher established that early administration of IV antibiotics like ceftriaxone helped in decreasing complications rates including; sepsis and preterm labor. Custodial care with at least 48 hours of hospitalization and continuing assessment of both mother and fetal status was deemed critical in achieving better results. Equally, Angelescu et al^[24]^ offered a systematic review of antibiotic treatment for UTIs during pregnancy. The meta-analysis of randomized controlled trials suggested that penicillins and cephalosporins were effective in eliminating UTIs and avoiding adverse fetal effects. Tackling asymptomatic bacteriuria was also stressed in this study because the failure to manage it leads to pyelonephritis and adverse maternal and fetal outcomes such as preterm delivery and low birth weight.^[107]^ Mørkved et al^[29]^ have also carried out another relevant clinical trial aimed at preventing urinary incontinence during pregnancy. The evidence presented showed that PFMT as a nonpharmacological and proven intervention compared to medications reduced incontinence incidents among pregnant and postpartum women. One major point of the study was that PFMT should be carried out at the initial stage to avoid aggravation of bladder dysfunction.^[108]^

### 2.46. Analysis of case studies highlighting successful interventions

Real-life scenarios elaborating pharmacological management during pregnancy are explained further in case studies. An example in this context is a 32-year-old pregnant female having recurrent UTI and a history of resistance to antibiotics.^[109]^ When ordinary antibiotics were tried and proved ineffective, the patient began a culture-directed therapy using nitrofurantoin; this treatment was successful at removing the infection without harming the fetus in any way.^[109]^ Another case that Semins and Matlaga described is of chronic kidney stones complaint in a pregnant woman at twenty-six weeks of gestation. Initially, an expectant approach with plain fluids and analgesia was undertaken, but given the worsening symptoms, the patient underwent placement of ureteral stents under the local block.^[110]^ The procedure was effective in relieving obstruction which the patient had gone through and did not affect the fetus as the patient was able to deliver a healthy baby at term. In both cases, these galleries underscore the need to come up with a unique approach when dealing with pregnant women with urological complications mainly because they both started with pharmacological management before shifting to non-pharmacological measures.^[111]^

### 2.47. Evidence-based practices and emerging trends

More studies on pharmacological interventions during pregnancy stress a call for practice-based evidence. The last few years brought up some developments concerning urological problems during pregnancy; the utilization of culture-based antibiotics that help prevent the development of antibiotic resistance and the shift to non-drug treatments such as PFMT of bladder dysfunction.^[112]^ There are new trends too, for instance, the utilization of alpha-blockers including and especially tamsulosin for the treatment of nephrolithiasis during pregnancy with obstructive nephropathy.^[113]^ The emerging trends in patient-tailored management considering the unique presentation and genetic makeup of an individual offered a prospect for enhanced early identification and better control of the urological complications during pregnancy.^[111]^ Further studies on safer drugs with little effect on the fetus, the best regimen dose and duration, and the best management practices informed by the latest trial evidence will probably dominate future research.^[114]^

### 2.48. Ethical considerations in pharmacological treatment during pregnancy

The use of drugs during pregnancy has several ethical dilemmas compared to other persons since the 2 parties involved, i.e. the mother and the fetus, both have rights that need to be protected.^[115]^ The decision-making process for the physicians and the healthcare providers involves analyzing and comparing the benefits and the hazards of an intervention with the patient’s self-assumed and competent preferences.

### 2.49. Balancing maternal health with fetal safety

The first of them is the conflict of interest relating to the assessment of pregnant women that consists of the efforts to provide proper protection for a woman during her pregnancy without harming the potential of the fetus. In serious infections such as pyelonephritis, uncontrolled infection may result in devastating maternal consequences such as sepsis or preterm labor, which also pose a risk to the fetus.^[49]^ However, some medicines used for these ailments since they belong to category D or X can have implications on embryo advancement.^[116]^ Hence, according to the principle of beneficence where one is supposed to do what is best for the client, the healthcare provider will have to decide whether to give the mother a teratogenic drug such as fluoroquinolones and tetracycline which has the risk of causing neonatal infection or not give her any antibiotic at all, knowing that she might die from a major infection.^[117]^ In such situations, the choice must be made with input from the patient along with the severity of the condition, other treatments possible, and gestational age of the fetus.

### 2.50. Informed consent and patient autonomy

The principle of informed consent is one of the fundamental tenets of ethical practice in medicine especially in pregnancy given its impact on the mother and the fetus.^[118]^ It means that pregnant women must know the possible undesirable outcomes of any planned pharmacological intervention as well as the benefits that may be received; they have to undergo information about the possible teratogenic effects and possible consequences for the further development of the fetus. Before making this decision the patient must be fully informed of the possible complications related to treating her condition and not treating it at all is mandatory for patients’ rights and autonomy.^[119]^ It will be embarrassing for a pregnant woman to undergo treatment that may harm her fetus, even if her condition is dangerous to her life. Despite this, healthcare providers have to abide by her decision as they continue to provide counsel, follow up, and recommend other forms of treatment if any.^[120]^ The principle of autonomy also applies here to the woman through decisional authority in that every woman has the right to accept or refuse treatment.^[118]^

### 2.51. Ethical dilemmas in prescribing medications

It remains a common ethical predicament since most drugs are potentially toxic during pregnancy and data on some of them is extremely limited.^[121]^ This is most particularly true in the case of recently developed drugs or where there is a definite paucity of data regarding their use in pregnant women. Due to the rise of uncertainties, healthcare providers need to handle them and maintain ethical principles such as non-maleficence; it is unethical to harm and justice is unethical, it is unfair treatment.^[122]^ For example, a physician should decide whether to give a pregnant woman a medication for kidney stones or urinary tract infection that has been proven in non-pregnant women but its risk for use during pregnancy is not well known.^[123]^ There is also an ethical requirement for providers to give pregnant women access to effective treatment for such diseases as urinary tract infections among others for the welfare of both the mother and the child.^[124]^

### 2.52. Interdisciplinary approach to managing urological complications

Many urological complications may arise in such pregnant women and their management is best done consultatively with obstetricians, gynecologists, and a team of other caregivers to increase the chances of positive outcomes for both mother and baby. This approach is very important in making sure all aspects of care have been addressed reducing risk and maximizing the benefits of the treatment plans.^[125]^

### 2.53. Collaboration between obstetricians, urologists, and pharmacists

Older women with complex urological problems including urinary incontinence, recurrent urinary infections, renal calculi, and vesical dysfunction should be managed by cooperation between obstetricians, urologists, and pharmacologists.^[126]^ Every specialist contributes his or her knowledge to the pregnant patient management. In terms of specialization, obstetricians are primary midwives in charge of monitoring fetal development while the urologist assists in the diagnosis of urological disorders and various urological complications including and particularly where surgery or comprehensive management of stones is desirable.^[37]^ In ensuring medication safety and health risks of medication interactions, side effects and teratogenicity of some drugs pharmacists can play pivotal roles.^[24]^ Their knowledge is most valuable when it concerns the choice of antibiotic treatments or painkillers proposed for application in pregnant patients.^[127]^

### 2.54. Importance of a multidisciplinary team in treatment planning

The concept of a multi-disciplinary team is thus especially relevant in the management of such patient circumstances such as pregnant women with recurrent UTIs or obstructing renal stones. In such cases, members of the team from different specialties have to sit down and come up with a treatment plan that can accommodate the urological condition and at the same time protect the expectant mother’s life.^[49]^ The various caregivers in the health sector need to keep communicating and or coordinating with each other in assessing the evolution of the pregnancy as well as the effects of the treatment. For instance, in a case of acute pyelonephritis the obstetrician and urologist consult to assess whether hospital admission is required, subsequent intravenous antibiotic treatment, or if surgery is required. In the meantime, the pharmacist checks all the recommended drugs to make sure that they are safe for pregnancy, in case proposing a dosage change or changing the medications.^[127]^ This strategy reduces risk factors and enhances baby-mother health outcomes during the delivery process.

### 2.55. Strategies for effective communication and coordination of care

In addressing the above urological complications during pregnancy, the members of the healthcare team should be very sensitive while sharing information. Thus, EHRs and shared treatment plans about the patient, her illness, the treatment she has undergone, and the results of her condition, can be shared among all the healthcare team members,.^[29]^ It also involves holding case conferences and team meetings where case issues and management decisions are reviewed and made. For instance, when treating bladder dysfunction during pregnancy, the obstetrician, urologist, and physical therapist work hand in hand to ensure that non-pharmacological interventions including pelvic floor muscle training are incorporated into the management plan of the patient.^[128]^ Medications that may be indicated for a patient, if necessary, are taken utilizing the patient’s medication record and the possible risk to fetal development.^[129]^ In general, the approach implies that the patient gets complete care of the urological condition and the issues of pregnancy.

### 2.56. Ethical considerations in pharmacological treatment during pregnancy

A key component of the pharmacological treatment of urological complications during pregnancy is ethical considerations as the choice of treatment has an impact on both the mother as well as the unborn child.^[130]^ Principles that govern good practice when making such decisions include: respect for the autonomy of the patient, do not harm, and the principle of beneficence as well as the principle of justice.^[131]^

### 2.57. Balancing maternal health with fetal safety

Use of drugs during pregnancy may entail consideration of the risk to the mother against the risks posed to the developing fetus. This conundrum is more apparent during treatment for severe diseases such as pyelonephritis, which may result in complications for the mother including sepsis or preterm labor.^[49]^ Guided by the principle of beneficence, the treatment of pregnant women requires clinicians to balance the treatment’s ability to help the pregnant woman against the potential risks that the treatment poses to the unborn child.^[132]^ Specifically, initial evidence abounds that tetracyclines and fluoroquinolones are teratogenic; thus, clients taking these medications are usually warned of the risks involved.^[24]^ Indeed, clinicians are advised to consider other approaches whenever possible, for example, non-medication management of renal lithiasis and lower urinary tract dysfunction withholding medication use potentially risky for the fetus.^[132]^ However, when the mother’s life is in danger, powerful drugs may be needed since severe maternal disease is also dangerous for fetal development.^[133]^

### 2.58. Informed consent and patient autonomy

This principle of autonomy postulates that pregnant women should make their own decisions on their treatment without undue influence from others.^[134]^ Such pregnant patients must be able to appreciate the consequences of the intended pharmacological therapies and other options.^[135]^ Still, healthcare providers need to communicate these risks in a manner that is easy to understand and then the patient can make an educated choice based on her values, beliefs, and preferences.^[136]^ Sometimes a pregnant woman does not agree to pharmacological therapy due to the possible negative impact on the fetus.^[137]^ For example, a woman with recurrent urinary tract infections may decide to forgo antibiotic treatment even though she would be at risk for pyelonephritis. In such circumstances, the clinician may have to bow to the patient’s verdict but at the same time not relinquish the role of counseling patients and explaining the other possible courses of action that are available. The best-known principle of respect autonomy intends to recognize the patient’s ability to make decisions on matters concerning her body, despite the negative impacts on her health.^[138]^

### 2.59. Ethical dilemmas in prescribing medications

There are usually ethical challenges in prescribing drugs to pregnant women because there are few studies about drugs and their effects on pregnancy. The non-maleficence principle means that a clinician should not provide treatments that can harm the fetus unnecessarily.^[139]^ However, there are circumstances when clinicians have to make decisions in conditions of uncertainty or contradiction, in other words, when the interest of the mother has to be balanced against the potential risks of the fetus. For example, an alpha-blocker like tamsulosin used in the management of renal calculi is not safe in pregnancy and there is limited literature to support the same.^[140]^ In such cases, there is a difficult balancing act in which often only a part of the disease process is treated using absolutely minimum of drugs possible because of contraindications or the possibility of side effects.^[141]^ Another ethical issue can be in cases when otherwise, pregnancy unsafe medications are the most effective in treating a life-threatening illness that the mother is suffering from. In these circumstances, the principle of justice, which is fundamental to providing equal access to effective health care, must be applied to guarantee pregnant women the right care that they need.^[142]^

### 2.60. Interdisciplinary approach to managing urological complications

Many urological complications during pregnancy are best treated with the help of synchronized action of an obstetrician, a urologist, a pharmacologist, and any other specialist who will be able to contribute their talents toward solving the problem. This makes the program effective in its results of having both maternal as well as fetal benefits.^[143]^

### 2.61. Collaboration between obstetricians, urologists, and pharmacists

Those conditions include recurrent urinary tract infections, kidney stones, and bladder dysfunction, all of which can be addressed by various urological specialists during pregnancy. Specialists in obstetrics are concerned with fetal development and the effect that treatment may have on pregnancy, and specialists in urology are concerned with the initial pathological condition. The pharmacists play an important role by weighing the drug interactions, modifying the doses, and approving the use of medications, especially during occasions when a patient requires multiple prescriptions.^[144]^ For instance, in the treatment of acute pyelonephritis, coordination with an obstetrician and urologist is crucial to deciding on hospital admission and the choice of intravenous antibiotics.^[49]^ Pharmacists help by checking for compatibility of the antibiotics with pregnancy and suggesting substitutes when the prescribed antibiotics are unsafe in pregnancy.^[24]^ This makes sure that the needs of the mother are well met while at the same time avoiding complications in the baby.

### 2.62. Importance of a multidisciplinary team in treatment planning

Particularly, a multimodal treatment plan is often crucial for patients with serious conditions, for instance, obstructive nephropathy or recurrent urinary tract infection in pregnancy.^[145]^ In these cases, consultation among obstetricians and urologists, nephrologists, and pharmacists is important in devising a treatment strategy that must take into consideration the urological disorder and the advancement of pregnancy.^[146]^ For example, the treatment of renal calculi in pregnancy mainly involves a conservative approach, with the urologist managing hydration and pain and the obstetrician managing fetal status.^[147]^ Occasionally, emergent procedures, including ureteral stenting, might be necessary, and the role of anesthesiologists and obstetricians is obligatory because pregnant women may have some specific anesthetic requirements.^[148]^ It’s important for all medications needed by pregnant women with urological conditions to be safe and effective and this is where pharmacists come in especially when the expectant women will be requiring more than one medication to treat both the urological and obstetric complications.^[149]^

### 2.63. Strategies for effective communication and coordination of care

Interprofessional collaboration is critical to the identification of urological issues and management of urological complications that may occur during pregnancy. EHRs and shared care plans can help all the members of the team to be aware of all the changes in the patient’s treatment and health status.^[29]^ There are also important that are case conferences and multidisciplinary team meetings the least frequent of which are necessary for discussing the cases that are considered complicated and making treatment decisions together.^[150]^ For instance, in managing UI in pregnancy, there is teamwork between obstetricians, urologists, and physical therapists to include treatment modalities such as pelvic floor muscle training in the management plan. This also ensures a systematized and integrated approach in the handling of urological disorders in addition to the well-being of the expecting mother and the fetus. Consequently, integrated teamwork is important to ensure proper urological evaluations are carried out during pregnancy.^[151]^ This kind of partnership between health care providers makes sure that both the life of the mother and that of the unborn fetus are protected and individualized on the various complications that come with pregnancy.

### 2.64. Non pharmacological interventions for UTIs during pregnancy

**Figure 4.**
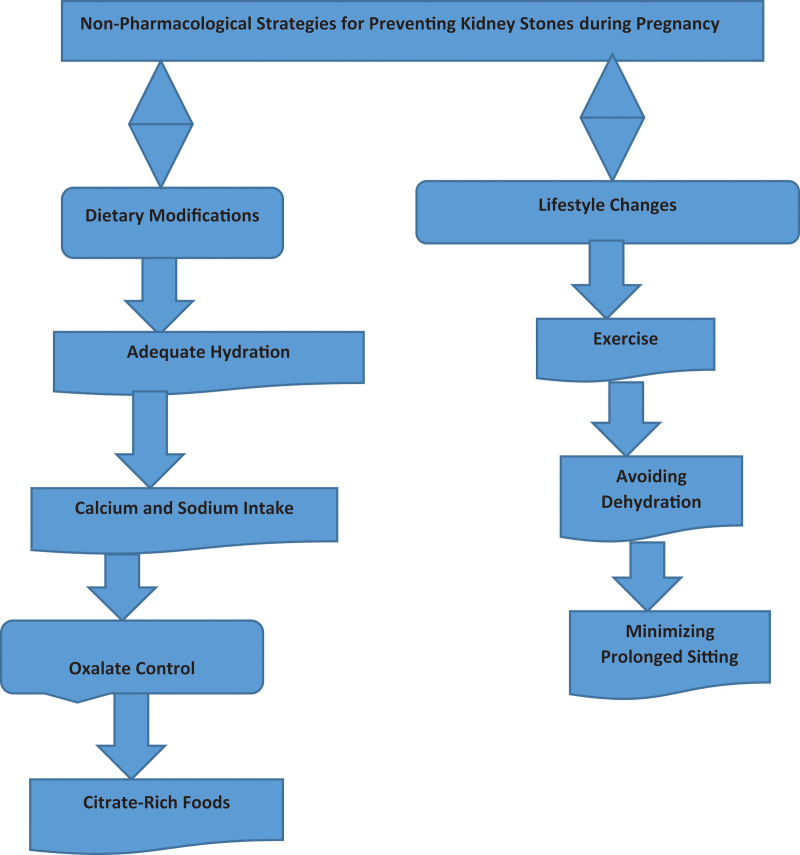
Non-pharmacological strategies for preventing kidney stones during pregnancy.

Antibiotic therapy is an important treatment for urinary tract infections (UTIs) during pregnancy, but non pharmacological interventions are also important in the management and prevention of these infections as shown in Figure 4.^[152]^ These will help to lower the chance of having a UTIs and can overall contribute to better bladder health during pregnancy.^[153,154]^

### 2.65. Increased fluid intake

One of the easiest and most effective ways to avoid UTIs is to drink enough fluids. If you’re a well hydrated bladder, you urinate more often, which acts as a cleansing mechanism to rid the urinary tract of bacteria and prevent bacteria that can get a strain foothold.^[153]^ Liquids are recommended to drink a lot but pregnant women are often advised to drink 8 to 10 cups of water a day but it depend on one’s level of activity, climate and other facts. Drinking more fluids will dilute your urine, and this may help protect your bladder, and flush out any harmful pathogens before they can cause infection.^[154]^ We need to drink water at all times (not just to relieve thirst), especially after meals and prior to and after physical activity. You may also want to avoid caffeinated beverages which may irritate the bladder.^[153]^

### 2.66. Frequent voiding

Frequent urination is a big preventative measure for UTIs.^[152]^ Women also want to be sure to empty their bladder regularly, such as right after every 2 to 4 hours but certainly not to go extended periods without releasing.^[153]^ By voiding frequently, urine doesn’t stagnate in the bladder as often, decreasing the chance bacteria will multiply. It is particularly so during pregnancy, when anatomical and hormonal changes can slow the voiding process.^[154]^ Even if you don’t have a strong feeling that you have to go to the bathroom, pregnant women must take care to not hold in your pee and delay urination.^[152]^ It can also help to empty the bladder before sleep if cases of nocturnal UTIs are to be avoided.

### 2.67. Proper hygiene practices

Proper hygiene is essential to prevent UTIs. It means wiping front to back after using the toilet and avoiding the transfer of bacteria from the rectum to the urethra.^[152]^ Another factor is avoiding the use of irritating products, such as douches, scented soaps and feminine sprays that could introduce harmful chemicals into the genital area upsetting the natural balance of vaginal flora.^[153]^ There’s no need to tell pregnant women to sleep on their backs, but they should be encouraged to wear cotton underwear, which will aid in ventilation and reduce moisture so that bacteria has less opportunity to thrive.^[154]^

### 2.68. Cranberry products

Although cranberry products (e.g. cranberry juices or supplements) are frequently recommended as adjunct therapy for the prevention of UTIs, research as to their effectiveness is mixed 158. Proanthocyanidins in cranberries may inhibit bacteria from sticking to the lining of the urinary tract, decreasing your chance of infection.^[153]^ While some studies have shown a benefit there is concern about consuming large amounts of cranberry juice in pregnancy as it does contain sugar, meaning it could cause other issues in pregnancy.^[154]^ Supplements containing cranberry should only be taken under the direction of a health professional.

### 2.69. Preventive measures for kidney stones during pregnancy

Complications that occur less often during pregnancy include kidney stones.^[155]^ The prevention of kidney stones is important in the management of maternal and fetal health, and there are several non pharmacologic strategies to reduce risk.^[152]^

### 2.70. Dietary modifications

Diet changes may play a big role in preventing or managing kidney stones during pregnancy.^[155]^ Some foods and nutrients that can lead to stone formation are identified and others that may help prevent it. Similar to UTIs, drinking adequate fluids helps prevent illnesses such as kidney stones.^[156]^ The higher the intake of water – the more diluted the urine – the less likely stone formation.^[156]^ Target amounts for urine produced by pregnant women are about 2.5 to 3L a day to prevent having stone formation as the less concentrated the urine is.^[157]^ Naturally, calcium based stones can form when calcium excretion in urine increases because of high sodium levels. Reducing the amount of salt you consume may help lower this risk. While calcium is good for bone health, taking too much calcium through supplements rather than from food can raise the risk of calcium stones.^[158]^ Calcium should be met through food sources (dairy and leafy greens) and fortified plant based alternatives, but avoid high dose calcium supplements unless on the advise of a healthcare provider. Eating foods high in oxalates (like spinach, beets, chocolate, and nuts) can increase the risk for forming calcium oxalate stones.^[159]^ Oxalates aren’t unhealthy in small amounts, but big boluses can increase your chances of stones, so in that case, it could be good to cut back on them.^[158]^ Because of that, lemons, oranges, and other citrusy fruits are rich in citrates, which can help prevent stone formation; when the citrates bind to calcium, crystals cannot form.^[159]^ Drinking lemon water or lemon water with juice from lemons and other citrus fruits for kidney stone prevention may also be helpful.^[159]^

### 2.71. Lifestyle changes

Certain lifestyle changes also help prevent kidney stones.

Exercise: Physical activity on a regular basis can help both maintain a healthy weight and keep the kidneys in good health. Obese or overweight is associated to high risk of getting kidney stones, so it is better to maintain a healthy body weight with regular exercise and eating a balanced diet.^[154]^

Avoid Dehydration: One primary risk factor for kidney stones is dehydration. Women who are pregnant should make sure to drink enough, especially in hot weather or with physical activity.^[155]^

Avoiding Prolonged Sitting: Continuous sitting is particularly important to be avoided for women at risk for kidney stones or who have had them before. Sitting for long periods can slow down the flow of your urine and also slow down your kidneys, which can increase your risk of forming a stone.^[156]^

## 3. Findings

Here are some novel findings and insights derived from this research on urological complications during pregnancy:

Increased Screening Recommendations: The incidence rate of UTIs ranges from 5–10% and bladder dysfunction from 20–50% so early screening may decrease the risk of acute pyelonephritis and preterm labor in the patient.

Role of Hormonal Changes: The review also shows that elevated levels of progesterone cause the smooth muscles of the urinary tract to relax. This finding might help in the development of specific approaches for the prevention of urinary retention and complications associated with incomplete voiding.

Pharmacological Innovation: A short-course antibiotic therapy for UTIs in pregnant women is less effective than the standard treatment regime owing to the altered physiology. Future studies could look into the best antibiotic protocols that have minimal risks to the mother and the baby.

Impact of Delayed Treatment: According to the review, the delayed treatment will worsen not only the health of the mother but also the development of the fetus. This means that early intervention measures should be put in place in cases of acute UTI or kidney stones to avoid the aforementioned complications such as low birth weight or sepsis.

Patient-Centric Management: As a patient-centered approach, the review highlights the ability of the personalized medicine to offer pharmacological treatments of urological complications based on patient’s risk profile to enhance the management of complications during the period of urological complications during pregnancy.

Bladder Dysfunction Treatment: Pelvic floor muscle training (PFMT) is a promising non-pharmacologic approach for addressing bladder dysfunction. This could be incorporated into prenatal care programs and could be recommended routinely for every woman of child bearing age.

A Model for Ethical Decision Making: The review presents an ethical decision making model for maternal patient and fetal patient based on autonomy. This systematic approach could complement the context of the clinician-patient conversations, especially in patients with multi-morbidities needing medications.

Cooperation Between Professionals of Different Fields: An integrated approach in the management of such cases requires the services of specialists such as obstetricians, urologists, and pharmacologists in cases of complications such as obstructive nephropathy or recurrent UTI. This collaboration could result in improved and safer management plans for the mother and the fetus.

Education and Awareness as the Main Goals: This research proposes that more awareness should be created on urological complications that may arise during pregnancy and how they should be handled. Lack of knowledge may be addressed through training programs that could also help healthcare providers follow updated guidelines.

### 3.1. Future research directions

According to this research, there is a call for more studies to determine safer pharmacological interventions with little or no adverse effects on the fetus, and the search for non-pharmacological options for pain and complications management during pregnancy. With our increasing understanding of maternal health and pregnancy complications, there are some important remaining research areas related to UTIs and kidney stones in pregnancy. Improvements in these areas hold the potential to inform better prevention strategies, increased treatment options, and a greater knowledge about long-term maternal and fetal outcomes.

#### 3.1.1. Development of novel antibiotics for UTIs

Antibiotics are important in UTI treatment, but antibiotics resistance in this infection is rising, especially in pregnant women who are easily attacked by infections. Investigating new classes of antibiotics or alternative therapies that get past resistant strains of bacteria, with minimum toxic effects to the mother and fetus, should be part of future research.

#### 3.1.2. Personalized medicine of UTI and kidney stone treatment

Pregnancy represents a dynamic physiological state one size does not fit all. Tailoring treatment plans according to one’s personal genetics, environment, and lifestyle can help prevent and manage UTIs and kidney stones during pregnancy. For instance, if there is a genetic predisposition to forming stones or recurrent UTI’s they can be identified early such that interventions can be more targeted.

#### 3.1.3. Preventive measures optimizing

There is further research required to fine tune preventative measures for UTI and kidney stones in pregnancy. More rigorous investigation of probiotics, cranberry products, or alternative therapies in the prevention of UTIs is needed. Other studies will further inform guidelines and recommendations for pregnant women regarding how different dietary changes, hydration strategies, and lifestyle changes can reduce their risk of developing kidney stones.

#### 3.1.4. Bladder and renal dysfunction in the long-term

Because of that, it’s crucial to understand the long term health implications of UTIs, kidney stones and bladder dysfunction during pregnancy, in order to improve maternal and fetal health. Long term research should be targeted at the effects these conditions have on mother’s kidney function, urinary health and risk of chronic conditions including hypertension, diabetes or chronic kidney disease. There should be more research on the long term neurodevelopmental outcomes of children born preterm or with low birth weight secondary to maternal infections or complications.

#### 3.1.5. Impact of novel lifestyle interventions

Clinical trials of innovative lifestyle modification approaches, such as diet, exercise, and hydration, are needed to explore how much they can help prevent UTIs and kidney stones in pregnancy. For instance, further work is currently needed to explore whether reducing intake of oxalates, or increasing intake of citrate, could have an impact on the incidence of kidney stones during pregnancy.

## 4. Conclusion

Urological disorders are common medical conditions that complicate pregnancy, including UTIs, kidney stones, and bladder dysfunction that may worsen both maternal and fetal well-being. Since pregnancy brings about changes in the physiology of the women, they are vulnerable to these conditions in their pregnancy and hence require very close monitoring and management. This review expands on the need to provide timely diagnosis and management to handle the progression of these problems to more severe stages with severe outcomes including preterm labor and decreased quality of life. Thus, this review, based on contemporary literature and clinical recommendations, insists on the use of protected interventions based on healthcare reform for both the infant and its mother.

### 4.1. Recommendations

Enhanced Screening and Monitoring: Screen pregnant women for urological complications regularly especially for women with this history of recurrent UTIs or kidney stones. Urinary cultures and Bladder function should be monitored on a more regular basis.

Education and Awareness: Notify healthcare providers of the high incidence and ways of handling complications involving the urinary bladder in pregnancy. Further, professional development programs may assist clinicians in adopting current guidelines and the best practices.

Individualized Treatment Plans: Ensure that treatment plans for all patients incorporate the patient’s history, symptoms, and the stage of pregnancy. This helps in developing intervention measures that have an impact on the safety and efficiency of operations.

Interdisciplinary Collaboration: Obstetricians, urologists, pharmacists, and many other people all work together like you and your friends do during group projects. Such synergism can make sure that the diverse care needs of patients are met and can enhance the probability of success in difficult cases.

Research and Development: Call outpatients and clinicians to continue to search for less risky pharmaceutical and auxiliary approaches like PFMT to manage urological complications during pregnancy.

Patient-Centered Care: Ensure that the patient’s preference guides the treatment process by informing the consent process. It appears that healthcare providers should ensure that risks and benefits of treatments are well explained to women so that they can participate in the decision-making process appropriately.

Guidelines and Protocols: Encourage the implementation and endorsement of definite clinical protocols on how to handle urological emergencies in pregnant women to increase overall compliance and raise the population’s health criteria in different centers.

## Acknowledgments

We are grateful to Kampala International University Uganda for its support.

## Author contributions

**Conceptualization:** Isaac Edyedu, Okechukwu Paul-Chima Ugwu, Chinyere N. Ugwu, Simeon Ikechukwu Egba.

**Methodology:** Isaac Edyedu, Okechukwu Paul-Chima Ugwu, Esther Ugo Alum, Val Hyginus Udoka Eze, Mariam Basajja, Jovita Nnenna Ugwu, Fabian Chukwudi Ogenyi, Regina Idu Ejemot-Nwadiaro.

**Supervision:** Okechukwu Paul-Chima Ugwu, Esther Ugo Alum, Val Hyginus Udoka Eze, Jovita Nnenna Ugwu, Regina Idu Ejemot-Nwadiaro, Simeon Ikechukwu Egba, Daniel Ejim Uti, Patrick Maduabuchi Aja.

**Validation:** Okechukwu Paul-Chima Ugwu, Chinyere N. Ugwu, Mariam Basajja, Jovita Nnenna Ugwu, Fabian Chukwudi Ogenyi, Michael Ben Okon, Simeon Ikechukwu Egba, Daniel Ejim Uti.

**Visualization:** Isaac Edyedu, Okechukwu Paul-Chima Ugwu, Mariam Basajja, Fabian Chukwudi Ogenyi, Michael Ben Okon, Simeon Ikechukwu Egba, Daniel Ejim Uti.

**Writing – original draft:** Isaac Edyedu, Okechukwu Paul-Chima Ugwu, Chinyere N. Ugwu, Esther Ugo Alum, Val Hyginus Udoka Eze, Mariam Basajja, Jovita Nnenna Ugwu, Fabian Chukwudi Ogenyi, Regina Idu Ejemot-Nwadiaro, Michael Ben Okon, Simeon Ikechukwu Egba, Daniel Ejim Uti, Patrick Maduabuchi Aja.

**Writing – review & editing:** Isaac Edyedu, Okechukwu Paul-Chima Ugwu, Chinyere N. Ugwu, Esther Ugo Alum, Val Hyginus Udoka Eze, Mariam Basajja, Jovita Nnenna Ugwu, Fabian Chukwudi Ogenyi, Regina Idu Ejemot-Nwadiaro, Michael Ben Okon, Simeon Ikechukwu Egba, Daniel Ejim Uti, Patrick Maduabuchi Aja.
